# Updating Clinical Practice: Improving Perioperative Pain Management for Adeno-Tonsillectomy in Children

**DOI:** 10.3390/children11101190

**Published:** 2024-09-29

**Authors:** Juan Manuel Redondo-Enríquez, María Rivas-Medina, Manuel María Galán-Mateos

**Affiliations:** Hospital de Mérida, 06800 Mérida, Badajoz, Spain; maria.rivasm@salud-juntaex.es (M.R.-M.); manuel.galan@salud-juntaex.es (M.M.G.-M.)

**Keywords:** acute postoperative pain, ibuprofen, NSAIDs, adenotonsillectomy, pediatric opioid-free anesthesia, preemptive analgesia, multimodal analgesia, Wong-Baker visual analog scale

## Abstract

Background/Objective: Perioperative acute pain management in pediatric patients is essential to reduce complications. Adenoidectomy-Tonsillectomy are surgical procedures requiring pain control, and risk minimization for postoperative bleeding, nausea, and vomiting. Despite their known secondary effects, the use of opioid analgesics is still preponderant in pediatric perioperative management. We performed a comprehensive review on adeno-tonsillectomy perioperative pain management in children. We developed and implemented a multimodal analgesia protocol aimed to improve patients’ pain management while consistently reducing opioids use. Methods/Results: relevant Information was summarized, then compared to our clinical needs. Learnings were used to create and implement a multimodal analgesia protocol that we use in patients 3–9 years-old undergoing adenoidectomy/tonsillectomy. The full protocol is presented. Analgesic strategies have emerged to reduce or avoid the use of opioids. Among these strategies, combining different non-opioid analgesics (Ibuprofen, Paracetamol, Metamizole) has been shown to be an effective and safe pharmacological strategy when implemented as part of perioperative multimodal analgesia protocols. Considerable evidence associating the use of NSAIDs with a bigger risk of postoperative bleeding does not exist. Conclusions: Perioperative management of adenotonsillectomy pain should include preventive and multimodal analgesia, which have shown to provide significantly more effective analgesia than some opioid regimens. Ibuprofen offers highly effective analgesia for postoperative pain, particularly when combined with acetaminophen.

## 1. Introduction

Pain is a complex and distressing experience both physically and emotionally, with actual or potential tissue damage, resulting from the interaction between neural pathways and neurochemical mediators. The perception of pain is highly personal; pain may have side effects on social and psychological function [1]. Pain management is essential to accelerate recovery by decreasing postoperative complications and comorbidities. Incorrect management favors long term complications such as chronic pain. The chronic pain prevalence is estimated to be 20–40% in the global population [2]. Patient’s age, sex and anxiety levels prior to surgery are some of the factors involved in the onset of acute pain [3,4]. Pain management in the pediatric population requires a bio-psychosocial approach to reduce patient’s anxiety when facing a surgical event [5].

Adenoidectomy and Tonsillectomy are some of most frequent surgical procedures in the pediatric population [6]. The two most common indications for tonsillectomy are recurrent throat infections and obstructive sleep-disordered breathing (oSDB). Surgery of the oral cavity usually produces intense pain and edema, which require quick action. Pain level may be directly associated to the surgical technique (extracapsular, intracapsular) and to whether cold or hot dissection is used [7]. The main cause of morbidity after tonsillectomy is oropharyngeal pain. A correct preoperative assessment decreases patient’s and family’s anxiety and stress before the procedure. Bleeding control, analgesia strategy, and nausea and vomit prevention are the main pillars in perioperative planning for the surgical team, and particularly for the anesthesiologist [8].

The treatment of postoperative acute pain in pediatric patients for these procedures is complex and could be inadequate in up to 30% of patients [2,3,9,10]. Interpatient variability in postoperative pain and the absence of adapted protocols often lead to patients being undertreated or overtreated [2,3,9]. Classically, opioid analgesics have represented the basis for the pharmacological treatment of perioperative acute pain in children. One of the main reasons for this might be the restricted availability of alternative non-opioid intravenous analgesic formulations. However, the systematic use of opioids and their incorrect administration schedule produce adverse events, whose frequency increases with higher opioid doses [11,12]. The most frequent opioid-related undesirable events are gastrointestinal (nausea and vomiting, constipation), neuropsychiatric, dermatologic (pruritus, rash), and central nervous system depression [13,14]. Also, opioids administered prior to sedation could significantly increase the risk of oxygen desaturation, the need for positive pressure ventilation (PPV), and vomiting [15]. Reducing the risk of opioid-related adverse events (AE) while providing adequate analgesia must be a clinical goal in perioperative pain management. More than twenty years ago, most analgesic protocols were based only on the control of postoperative pain. More recent analgesic strategies are designed to use multiple pharmacological and non-pharmacological procedures (Multimodal) targeting different pain pathways [11,12]. Today, there exist also multiple therapeutic strategies for multimodal analgesia included in Enhanced Recovery After Surgery (ERAS) programs or after Major Ambulatory Surgery (MAS). We aimed to perform an updated review on adeno-tonsillectomy perioperative pain management in children and use it to update our clinical practice through the development and implementation of a perioperative multimodal analgesia protocol aimed to improve adeno-tonsillectomy patients’ pain management while consistently reducing opioids use.

## 2. Search Methodology

We conducted a comprehensive review through online search on Medline for publications related to human beings aged 0–18 years including Controlled Clinical Trials, Metanalysis, Randomized Controlled Trials, and Systematic Reviews. Searched terms included: “pain” OR “acute postoperative pain” OR “NSAIDs”, OR “adenoidectomy” OR “tonsillectomy” OR “adenotonsillectomy” OR “ibuprofen” OR “opioid-free anesthesia” OR “analgesia” OR “fever” OR “preemptive analgesia” OR “multimodal analgesia” AND “pediatric” OR “children” OR “human” AND “controlled clinical trials” OR “metanalysis” OR “randomized controlled trials” AND (allchild[Filter]).

We conducted also online search on Medline for Protocols and Guidelines on Pediatric Analgesia (acute, UCI, surgery). Searched terms include: “analgesia” OR “pain” OR “adenoidectomy” OR “tonsillectomy” OR “adenotonsillectomy” AND “pediatric’ OR “children” AND “management guidelines” OR “practice guidelines”, AND (allchild[Filter]).

Additionally, for protocols and guidelines on pediatric analgesia we implemented also free-hand internet search.

**Figure 1:**
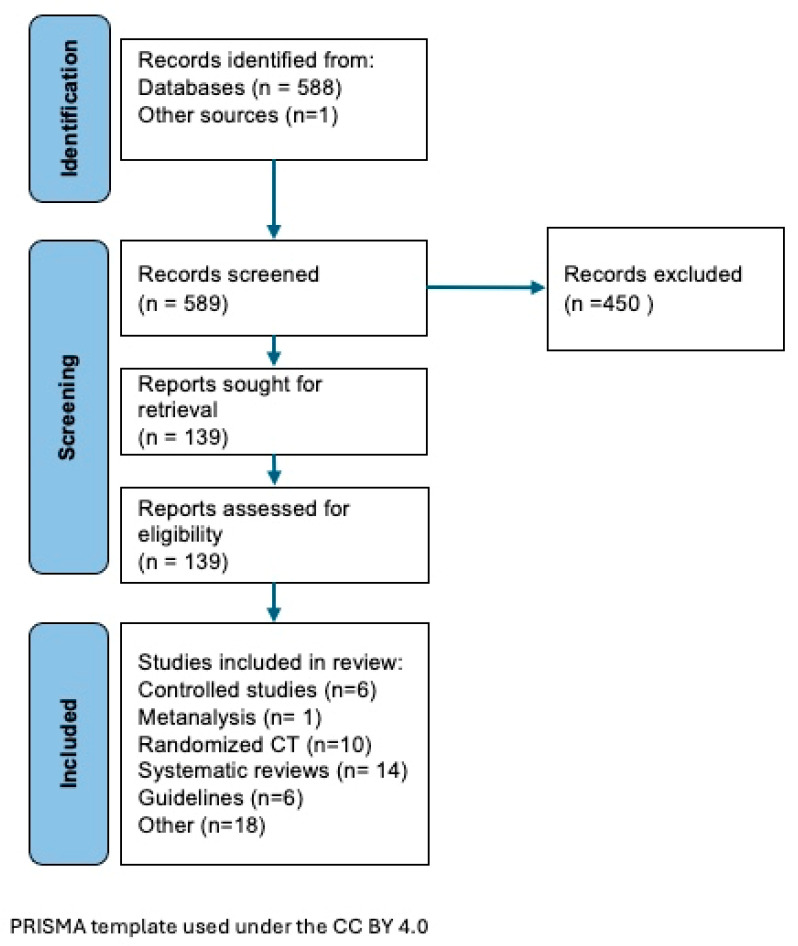
This figure was created using PRISMA recommendations and illustrate the type of published evidence that we finally considered for this article.

Out of an initial selection of 589 articles we considered 139 as possibly relevant and further reviewed them. A figure was created using the PRISMA recommendations (Figure 1) and is presented here to illustrate the type of published evidence that we finally considered for this article.

## 3. Findings and Discussion

There exist analgesic strategies involving the use of local anesthesia and adjuvants in the tonsillar bed [16], also, opioid-free analgesic (OFA) protocols rely on multiple adjuvant medications that target different pain receptors [17]. Commonly used pharmacological strategies include the use of paracetamol, metamizole, and non-steroidal anti-inflammatory drugs (NSAIDs) such as Ibuprofen. Monotherapy with paracetamol has been considered insufficient to control postoperative pain, thus paracetamol is used in combination with other medications [7].

Traditionally, oral NSAIDs have been helpful for the management in the pediatric population of certain clinical situations such as fever, inflammatory diseases, and postoperative pain [18]. Thanks to their anti-inflammatory effects, NSAIDs can play a significant role in multimodal analgesic strategies [19].

Perioperative administration of NSAIDs has been limited in case of hemostasis alterations because of their inhibitory action on Thromboxane A2 synthesis, which is responsible for a reduction of platelets activity [20]. The frequency of readmission for post-tonsillectomy bleeding is about 2–7%, and the reoperation rate for hemostasis is 1–2% [21]; its presence increases morbidity and hospitalization costs, and delays patient’s discharge from hospital. Preoperative risk factors include male sex, advanced age, previous history of abnormal bleeding, and the intake of platelet antiaggregants or other anticoagulants. However, several clinical studies on the subject have not shown any statistically significant relationship [21]. There exists only one retrospective cohort study putting in evidence a bleeding increase in patients older than 12 years with a history of recurrent tonsillitis [22]. In their published metanalysis, Geva et Brigger [23] concluded that it has not been possible to link the choice of a specific surgical technique to postoperative bleeding. Thus, the main factor to be assessed as possible cause of postoperative bleeding continues to be the medication therapy [24].

In their clinical practice guideline on tonsillectomy in children the American Academy of Otolaryngology-Head and Neck Surgery (AAO-HNS) concluded that NSAIDs are safe for pain control after tonsillectomy without significant increased risk for postoperative hemorrhage [25].

In children, Ibuprofen is the most widely assessed NSAID for pain management and can be used postoperatively from the age of 3 months. Ibuprofen is derived from the propionic acid, and has analgesic, anti-inflammatory and antipyretic properties. The main mechanism of action is based on the blockage of prostaglandins synthesis through the inhibition of the cyclooxygenase enzymes (COXs) [26]. The family of COX enzymes contribute to inflammation, fever, pain, coagulation and chemoprotection of the gastric mucosa. As a non-selective COX inhibitor, Ibuprofen can induce potential secondary effects associated to COX-1 inhibition, particularly in the urinary and gastrointestinal systems [27]. This inhibitory action of Ibuprofen towards COX isoforms is competitive and reversible. This reversibility allows full recovery of enzymatic activity upon metabolism and elimination of Ibuprofen, thus reducing the risk of side effects [28].

Furthermore, the classical clinical study from Lesko and Mitchell [29] did not show any significant safety differences between paracetamol and Ibuprofen when used as antipyretic in hospitalized patients. Ibuprofen can be administered orally, intravenously, and rectally.

When administered orally, 80% of Ibuprofen is absorbed in the gastrointestinal tract reaching the peak of plasma concentrations within 2 h of administration [30]. Ibuprofen shows a distribution volume of 0.1–0.2 L/kg, and an extensive binding (over 99%) to plasma proteins, particularly to albumin [31].

The metabolism of Ibuprofen is mostly hepatic, which involves hydroxylation and carboxylation of its Isobutyl group done through P450 cytochrome enzymatic complex as well as conjugation with guluronic acid. None of the metabolites have any pharmacological effect, nor have been found to be toxic. This means that there is no accumulation of active metabolites [32]. Ibuprofen can reach the Central Nervous System (CNS) and is present in free (i.e., non-protein bound) concentrations in the cerebrospinal fluid (CSF) [33]. Total urinary elimination of Ibuprofen, mostly under the form of inactive metabolites, happens within 24 h.

A recent literature review assessing the pharmacokinetics of NSAIDs in infants showed similar data for children and adults for both oral and IV routes [34]; and a more recent multicenter study found that the short-term safety profiles of IV ibuprofen in pediatric patients 1–6 months of age were comparable to those in children older than 6 months of age [35]. The recommended oral dosage in children is 5 to 10 mg/kg every 6–8 h with a maximum of 30–40 mg/kg/day [25].

The oral route is the most common administration route for Ibuprofen. The unavailability of IV NSAID such as IV Ibuprofen in operating rooms and recovery wards and the impossibility of using the oral route could be reasons for the more frequent use of IV opioids in the early postoperative period [36]. Thus, the administration of Ibuprofen has been limited to minor surgical procedures as only rectal suppositories could be used [32].

Traditionally in pediatrics, Ibuprofen has been used in the closure of the arterial duct in preterm new-born or in babies with low weight for gestational age. Ibuprofen has been authorized since 2004 by the European Medicines Agency (EMA) for the treatment of hemodynamically significant patent ductus arteriosus (PDA) in preterm new-born (less than 34 weeks of gestational age). Ibuprofen is not recommended for the management of fever or as an anti-inflammatory in children younger than 3 months and/or 5 kg of body weight.

Cramer et al. in their review [37] to support the AAO-NHS clinical practice guidelines on the opioid prescription for analgesia after common otolaryngology operations concluded that NSAIDs provide highly effective analgesia for postoperative pain, particularly when combined with acetaminophen, that inconsistent use of non opioid regimes arises from the misconception that NSAIDs are less potent than opioids and have an unacceptable risk of bleeding. Thus, the guideline prioritizes multimodal—non opioid analgesia as first-line therapy.

The FDA first approved in 2009 Ibuprofen’s intravenous presentation alone for the treatment of mild-to-moderate pain and combined to opioids to treat moderate-to-severe pain [38].

The Spanish Agency for Medicines and Medical Devices (AEMPS) hasn’t authorized so far, the use of Ibuprofen in children younger than 6 years or with a weight lower than 20 kg. However, based on available evidence its off-label use to manage mild-to-moderate postoperative pain in children is tolerated. Namely, the Spanish Society for Pediatric Intensive Care (SECIP) includes in its guidelines the off-label use of Ibuprofen as a first step in postoperative pain management, and such recommendation has been adopted in several Spanish hospitals’ pain protocols, as in Hospital Don Benito-Villanueva [39].

Findings from some clinical studies performed with ibuprofen in adults combined with current pharmacological knowledge were influential to define pain management strategies in children, along with the wide experience on pediatric pain and fever management with NSAIDs, mainly with ibuprofen.

A trial to assess the efficacy and safety of IV Ibuprofen vs. placebo for the management of postoperative pain, where all patients (*n* = 206) had access to morphine through a patient-controlled analgesia pump, found that the opioid requirements were significantly reduced by 48% in the group treated with ibuprofen (*p* < 0.01); VAS pain scores also decreased significantly (*p* < 0.01), and overall Ibuprofen was safe and well tolerated [40]. Similar results were found by other authors in different clinical situations: Kroll et al. in gynecological surgery [41], Singla et al. in orthopedic surgery [42], Bayouth et al. in traumatology [43], and Gupta et al. also in orthopedics [44].

Since 2001 Kelly et al. studied the efficacy of blocking pain transmission before the surgical incision is made. This concept called pre-emptive analgesia [45] includes three main objectives: to inhibit acute pain during and after surgery, modulate pain at nervous system level, and decrease the risk of postoperative chronic pain. Regarding ibuprofen, there is evidence indicating that a single IV dose, administered before the surgical incision is made, significantly decreases the postoperative need for opioids.

The influential work from Moss et al. published in 2014 showed that the preoperative administration of a single dose of IV Ibuprofen (10 mg/kg) to patients aged 6–17 years, significantly reduced fentanyl usage after tonsillectomy [46]. Peng et al. assessed preemptive analgesia in 40 children aged 9–24 months who underwent cleft palate repair and showed that preemptive IV Ibuprofen 10 mg/kg at induction had a significant opioid sparing effect [47]. Additional evidence on preemptive analgesia has been published by Ahiskalioglu et al. [48], Gozeler et al. [49], Le et al. [50], Demirbas et al. [51], Mutlu and Ince [52], and Viswanath et al. [53].

Although it can be considered that tonsillectomy produces at least moderate pain, post-tonsillectomy pain must be individually assessed. Traditionally, under the World Health Organization’s (WHO) pain management scale, post-tonsillectomy pain would require the combination of paracetamol or NSAID with minor opioids like tramadol or codeine. However, the clinical practice guidelines from the American Academy of Otolaryngology do not recommend the use of codeine post-tonsillectomy in children younger than 12 years [25]. Multiple regulatory authorities including the FDA, the MHRA, the EMA and the Australian TGA warn against using codeine in children and contraindicate its use following tonsillectomy [54].

Evidence about the effects of the addition of IV Ibuprofen to multimodal analgesia regimens in pediatric patients have been recently published. A randomized clinical trial including children aged 6 months to 6 years assessed analgesia protocols after surgical hernia repair. Patients were randomized to paracetamol alone (P), Ibuprofen alone (I) or to a combination of both medications (I + P). Only 12.8% of patients in the (I + P) group required rescue fentanyl, compared to 28.6% in the (I) group and 66.7% in the (P) group [55]. In another trial, sixty-eight patients aged 2–12 years who underwent open cardiac surgery were included and randomly allocated to ibuprofen or to placebo. Postoperative fentanyl consumption as well as pain scores were significantly lower in the Ibuprofen group [56]. In their recent Cochrane review, Pessano et al. [36] reported an increasing trend in perioperative use of intravenous ibuprofen in children, and for its general use for acute postoperative pain in children, they highlight a reduction in adverse events compared to morphine, and little to no difference in bleeding when compared to paracetamol.

Thus, the pre-emptive use of IV Ibuprofen and the combined use of paracetamol/Ibuprofen in postoperative pain management seem supported by the subsequent reduction in the total quantity of required opioids, which offers the potential benefit of a reduction in opioids-related adverse events.

### Creating a Pediatric Analgesia Strategy

We decided to implement our protocol as a multimodal strategy. The following steps included to determine the most appropriate medications and the best way to optimize their combined use while following relevant guidance and recommendations.

Our literature review highlighted the scarcity of efficacious and validated perioperative analgesia protocols particularly in children. Reported evidence is not homogeneous, which makes difficult to draw significant conclusions. Almost 90% of studies assessed oral analgesic formulations, however administering oral medications is possible only after anesthesia recovery is completed. As a result, IV formulations of non-opioid medications, which are clinically appropriate, should be readily available at operating and recovery rooms.

Children must benefit from an analgesic plan that is effective, safe, flexible, easy to implement, and tailored to their clinical condition. Available clinical evidence supports the combined use of analgesic techniques and medications before and after surgery. Multimodal analgesia should provide significantly more effective analgesia than some opioid-based regimens.

Our hospital provides tertiary care services; each year we perform 70–100 pediatric adenoidectomy/tonsillectomy procedures. This protocol is hence primarily intended to be used in children 3 to 9 years old undergoing adeno-tonsillectomy. So far, we have limited the use to this population simply because all our patient undergoing adeno-tonsillectomy are within this age range. The protocol in presented in full in Table 1.

The protocol we propose here for the perioperative management of pain after adeno-tonsillectomy in children 3–9 years old meets all these requirements and is easy to implement without increasing workload to clinical staff.

The proposed sequence of medications administration and doses has been created not only to benefit from the preventive analgesic action of Ibuprofen but also to reduce any risk of Ibuprofen overdose.

Early detection and appropriate perioperative management must be at the core of surgical pain management as these are currently quality criteria for clinical care. Pain in children should be regularly assessed using consistent and validated tools suitable for each patient’s situation.

Anesthesiologists must lead the way to increase awareness on the need for a suitable management of pain in children and accelerate the implementation of multimodal analgesia protocols.

Inconsistent use of nonopioid regimens might arise from common misconceptions that NSAIDs are less potent analgesics than opioids and have an unacceptable risk of bleeding.

We use morphine hydrochloride in the protocol only because our hospital decided to homogenize the use of IV opioids, however, any other suitable opioid such as fentanyl could be used instead [58].

We have noticed a reduction in the need for opioid rescue during the immediate postoperative period while patients are still in the recovery unit. Based on early empirical observations we believe that this protocol could be considered successful when only 10% or less of patients require morphine hydrochloride rescue during their stay at the post-anesthesia recovery unit. This figure is in line with those presented by Lee et al. [55] who reported that only 12.8% of children in the group receiving a combination of Ibuprofen plus Paracetamol after inguinal hernia surgery needed rescue fentanyl.

We have not seen any increase in readmissions rates due to pain or bleeding during the first ten postoperative days.

## 4. Conclusions

Perioperative pain management strategies in children including pre-emptive analgesia and multimodal pain management protocols seem to support quicker recovery, increase patient comfort and reduce postoperative complications, which seems to be linked to a reduction or avoidance of opioids use.

NSAIDs, in this case IV Ibuprofen used immediately before surgery and postoperatively in combination with other non-opioid analgesics, such as Paracetamol, represent a helpful tool for the implementation of perioperative analgesia strategies.

## 5. Future Directions

The development of new medicines, the increased availability of new formulations more adapted to perioperative situations, the creation of multimodal analgesia protocols, the use of protocols with reduced or absent opioids, and the discovery of new local and regional analgesia techniques are some of the strategies aimed to reduce acute and chronic pain, and to improve patients’ quality of life. This approach to pain management could certainly be helpful for all types of surgery in children.

Hopefully, pre-emptive and multimodal analgesia will become generalized, and it is important to keep in mind that minimal changes to the way we use tools that have been available for decades, i.e., pre-emptive use of Ibuprofen, could result in significant clinical outcomes improvement.

Clearly, clinical evidence will be required to accelerate improvements in pediatric pain management, and to ease and support their adoption by the largest possible number of institutions and practitioners. Further assessment of this protocol would be needed to confirm our first observations. A formal clinical study will be the next logical step.

## Figures and Tables

**Table 1:** Adeno-tonsillectomy in children 3–9 years. Multimodal protocol for pain management.

Surgical Stage	Anesthesia Activity	Analgesia Activity	Medication	Dose/Route	Comments
Preoperative	Anxiolysis		Midazolam	0.05–0.1 mg/kg IV	Administered immediately before transporting the patient to the OR
	Anesthesia induction		Fentanyl	1–2 mcg/kg IV	Performed after standard basic monitoring and pre-oxygenation, followed by endotracheal intubation and connection to mechanical ventilation
			Propofol	2–3 mg/kg IV	
			Rocuronium	0.6–1.2 mg/kg IV	
	Antiemetic prophylaxis		Dexamethasone	0.15 mg/kg IV and	Double antiemetic prophylaxis
			Ondansetron	0.15 mg/kg IV	
		Pre-emptive analgesia [34,44,45,46,47,48,49,50]	Ibuprofen	10 mg/kg IV single dose	Performed before any surgical action or incision is made
Intraoperative	Anesthesia-Maintenance		Choice of: Propofol or	IV infusion, 9–15 mg/kg/h	Propofol reduces the incidence of post operative nausea and vomiting, which can be an advantage in ambulatory surgery. Sevoflurane provides bronchodilation, can be a better choice in patients with asthma.
			Sevoflurane	Vaporizer calibrated to provide 2% MAC (in Oxygen)	
		Rescue Analgesia	Paracetamol [35,37]	10–15 mg/kg IV	
Postoperative	On surgery completion, during the immediate postoperative period and after neuromuscular blockade reversal and patient’s extubation, analgesia monitoring is performed every 30 min using the Wong-Baker visual analog scale (WBS) [57].				
		Pain assessment WBS 2–3	Metamizole	20–30 mg/kg IV	Single dose. Risk of agranulocytosis
		IF pain evolves to moderate or severe (WBS > 4)	Morphine hydrochloride	0.05–0.1 mg/kg IV	Single dose.
		Followed by rescue analgesia [25,53]	Alternating -Paracetamol	10 mg/kg every 8 h, IV then PO	Making sure they are tolerated orally before patient is sent home.
			-Ibuprofen [34]	10 mg/kg every 8 h IV then PO	
